# Fungal-host interactions: insights into microRNA in response to *Paracoccidioides* species

**DOI:** 10.1590/0074-02760200238

**Published:** 2020-08-03

**Authors:** Junya de Lacorte Singulani, Julhiany de Fátima da Silva, Fernanda Patricia Gullo, Marina Célia Costa, Ana Marisa Fusco-Almeida, Francisco Javier Enguita, Maria José Soares Mendes-Giannini

**Affiliations:** 1 Universidade Estadual Paulista, Faculdade de Ciências Farmacêuticas, Araraquara, SP, Brasil; 2 Universidade de Lisboa, Faculdade de Medicina, Instituto de Medicina Molecular, Lisboa, Portugal

**Keywords:** paracoccidioidomycosis, microRNAs, pathways, target genes, lung cells

## Abstract

**BACKGROUND:**

*Paracoccidioides* spp. causes paracoccidioidomycosis (PCM), an important and frequent systemic mycosis that occurs in Latin America. The infectious process begins with contact between the fungus and lung cells, and the molecular pattern of this interaction is currently poorly understood. MicroRNAs (miRNAs) are small non-coding RNAs that regulate the gene expression in many biological processes, including in the infections.

**OBJECTIVE:**

This study aimed to analyse the expression of miRNAs in lung cells as response to infection by *Paracoccidioides* spp.

**METHODS:**

A quantitative real-time polymerase chain reaction (RT-qPCR) based screening was employed to verify differentially expressed miRNAs in human lung cells infected with three different species; *Paracoccidioides lutzii*, *Paracoccidioides americana*, and *Paracoccidioides brasiliensis*. Furthermore, the *in silico* predictions of target genes and pathways for miRNAs were obtained.

**FINDINGS:**

The results showed that miRNAs identified in the lung cells were different according to the species studied. However, based on the predicted targets, the potential signaling pathways regulated by miRNAs are common and related to adhesion, actin cytoskeleton rearrangement, apoptosis, and immune response mediated by T cells and TGF-β.

**MAIN CONCLUSIONS:**

In summary, this study showed the miRNAs pattern of epithelial cells in response to infection by *Paracoccidioides* species and the potential role of these molecules in the regulation of key pathogenesis mechanisms of PCM.

Paracoccidioidomycosis (PCM) is a systemic mycosis, endemic in Latin America. This mycosis is acquired after inhalation of conidia of dimorphic fungus *Paracoccidioides* spp. and the infectious process begins in the lungs of patients, but the fungus can spread to other organs.^1^ The *Paracoccidioides* genus is composed of a clade with five phylogenetic species: *Paracoccidioides brasiliensis* (S1a, S1b, PS2, PS3, and PS4) and a *Paracoccidioides lutzii* clade.^2^ ^,^ ^3^ Recently, a new taxonomic description was suggested, wherein phylogenetic species PS2, PS3, and PS4 were reclassified as species *Paracoccidioides americana*, *Paracoccidioides restrepiensis* and *Paracoccidioides venezuelensis*, respectively.^4^ In this context, the correct molecular taxonomy of these fungi has opened new possibilities for studying and understanding their relationships with their hosts.^5^

MicroRNAs or miRNAs are small non-coding RNAs with approximately 22 nucleotides that are expressed by all metazoan eukaryotes and have emerged as major controllers of gene expression of at least 30% of human genes. They mediate the regulation of gene expression in response to pathogen infection and are known to be the primary regulators of an immune response.^6^ ^,^ ^7^ ^,^ ^8^ In this context, differential expression of miRNAs has been described in response to infections by fungi such as *Candida albicans*,^9^ *Aspergillus fumigatus*,^10^ and *Cryptococcus neoformans*.^11^

In previous studies, the expressions of miRNAs in the lung tissues of mice infected by *P. brasiliensis* ^12^ and in the serum of patients with PCM^13^ were analysed. In this study, we analysed the expression of miRNAs directly in human lung cells in response to infection by different species of the genus *Paracoccidioides*.

## MATERIALS AND METHODS

*Infection and extraction of RNAs* - Three species; *P. lutzii* 01 (ATCC MYA-826, Goiânia), *P. americana* 02 (Venezuela), and *P. brasiliensis* 18 (São Paulo) from the collection of the Laboratory of Clinical Mycology, Faculty of Pharmaceutical Sciences of UNESP, Araraquara (Brazil) were used in this study. The fungi were maintained in Fava-Netto medium at 37ºC and were used after three to four days of growth.

The human A549 lung carcinoma cell line was obtained commercially (Code: 0033; Banco de células do Rio de Janeiro - BCRJ, Federal University of Rio de Janeiro, Brazil). The cells were maintained in HAM F-12 medium, supplemented with 10% fetal bovine serum at 37ºC in a 5% CO_2_ atmosphere.

For the experiments, cells (adjusted to 10^4^ cells/mL) were grown in 75 mm^3^ cell culture flasks for 24 h in HAM F-12 medium supplemented with 10% foetal bovine serum at 37ºC and 5% CO_2_ to reach at least 80% confluence. Then, lung cells were incubated with 10^6^ cells/mL of each fungal species (multiplicity of infection - MOI 100:1) at 37ºC for 5 h. After, the cells were washed with phosphate-buffered saline (PBS) to remove the unbound fungi. Uninfected lung cells were used as control. Samples were immediately frozen at -80ºC. The experiments were carried out in triplicate, and at least three independent experiments were performed.

The extraction of RNA was performed using Trizol (Invitrogen) followed by purification using the RNeasy Mini Kit (Qiagen) according to the manufacturer’s instructions. The concentration of total RNA was determined at 260 nm using NanoDrop 2000/2000c and the RNA integrity was analysed using a Bioanalyzer Agilent 2100.

*Complementary DNA (cDNA) synthesis and quantitative real-time polymerase chain reaction (RT-qPCR)* - cDNA synthesis of mRNA was performed with Universal a cDNA Synthesis kit (Exiqon A/S) according to the manufacturer’s instructions. After, quantification of miRNAs was performed by RT-qPCR with SYBR - Green PCR Master Mix (Exiqon A/S) at a total reaction volume of 80 µL. Then, 10 µL of the reaction mixture was added to each well of a panel (microRNA Ready-to-Use PCR, Human panel I+II, V.2 M/R; Exiqon A/S), which contained specific primers for 752 unique human miRNAs. Amplification was performed using a ViiA™ 7 Real-Time PCR System (Thermo Fisher Scientific, Inc., Waltham, MA, USA) followed by the determination of the melting curve. Cycling conditions were 95ºC for 10 min followed by 40 cycles of 95ºC for 10 s and 60ºC for 60 s. Expression profiling of miRNAs was calculated using the ΔΔCt method,^14^ with ΔΔCt as the difference between infected and non-infected cells. The endogenous controls UniSp6, U6 snRNA, SNORD38B and SNORD49A were used to normalise the analysis in each sample. Statistical analysis was performed using DataAssist v2 Software (Applied Biosystems; Thermo Fisher Scientific, Inc.) and p < 0.05 was considered statistically significant.

*Pathways analysis* - Predicted miRNA targets and potential infection pathways for miRNAs were obtained using the DIANA miRNA and mirPath tools^15^ and functional KEGG pathway database.^16^

## RESULTS

In this study, several miRNAs among 752 human miRNAs analysed were differentially expressed in A459 lung cells after infection with *Paracoccidioides* spp. A period of 5 h of infection was selected because a previous study demonstrated a higher percentage of infected A549 cells by *P. brasiliensis* at this time compared to others (2, 18, 24 and 48 h). In addition, after 5 h, the process of apoptosis in A549 cells gradually increased up to 48 h, reducing the cell viability.^17^ The data generated by RT-qPCR (p < 0.05 and fold-change ≥ 2) showed that miRNAs expressed in lung cells were different according to the phylogenetic species of the fungus. Most miRNAs (8) were downregulated in the cells infected with *P. lutzii* (Pl01) compared to non-infected cells (Table I). On the other hand, most miRNAs (21) were up regulated in infected cells with *P. americana* 02 (Pa02) compared to the control (Table II). In the cells infected with *P. brasiliensis* 18 (Pb18), eight miRNAs were up-regulated and 10 miRNAs down-regulated compared to the control (Table III).

**TABLE I:** Differentially expressed miRNAs in human lung cells infected with *Paracoccidioides lutzii* (Pl01)

Upregulated	Fold change	p value
miR-1911-3p	2.12	0.0496
Downregulated	Fold change	p value
miR-940	0.55	0.0275
miR-26b-5p	0.52	0.0422
miR-379-5p	0.49	0.0020
miR-146a-5p	0.47	0.0104
miR-17-5p	0.41	0.0178
miR-185-5p	0.39	0.0304
miR-454-3p	0.31	0.0441
miR-24-1-5p	0.29	0.0331

**TABLE II:** Differentially expressed miRNAs in human lung cells infected with *Paracoccidioides americana* 02 (Pa02)

Upregulated	Fold change	p value
miR-92a-1-5p	5.80	0.0075
miR-20a-3p	3.47	0.0076
miR-125b-1-3p	2.85	0.0342
miR-181a-3p	2.83	0.0305
miR-125a-3p	2.63	0.0224
miR-941	2.60	0.0053
miR-542-5p	2.52	0.0345
miR-1245	2.45	0.0265
miR-18a-5p	2.44	0.0343
miR-187-3p	2.37	0.0262
miR-639	2.27	0.0322
miR-506-3p	2.27	0.0322
miR-195-3p	2.26	0.0216
miR-153	2.24	0.0332
miR-432-3p	2.21	0.0040
miR-200a-3p	2.20	0.0395
miR-1267	2.20	0.0402
miR-769-5p	2.17	0.0148
miR-7-2-3p	2.13	0.0428
miR-936	2.11	0.0284
miR-450b-5p	2.07	0.0118
Downregulated	Fold change	p value
miR-9-5p	0.47	0.0341

**TABLE III:** Differentially expressed miRNAs in human lung cells infected with *Paracoccidioides brasiliensis* 18 (Pb18)

Upregulated	Fold change	p value
miR-26b-3p	3.81	0.0324
miR-27a-5p	3.50	0.0388
miR-92a-1-5p	3.43	0.0015
miR-149-3p	3.17	0.0071
miR-550a-3p	2.40	0.0109
miR-145-5p	2.35	0.0030
miR-181a-2-3p	2.34	0.0209
miR-941	2.24	0.0293
Downregulated	Fold change	p value
miR-331-3p	0.53	0.0431
miR-27a-3p	0.50	0.0185
miR-185-5p	0.44	0.0215
miR-30a-5p	0.43	0.0301
miR-379-5p	0.42	0.0118
miR-26b-5p	0.42	0.0031
miR-590-5p	0.35	0.0338
miR-30e-5p	0.34	0.0381
miR-324-5p	0.22	0.0371
miR-1538	0.15	0.0473

**Fig. 1:**
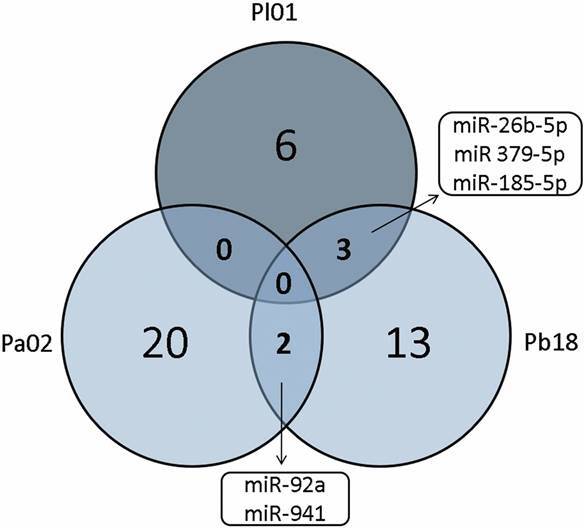
cell miRNAs following fungal exposure. Venn diagram with common miRNAs in A549 cells infected with the different phylogenetic lineages of the genus *Paracoccidioides*; *P. lutzii* 01 (Pl01), *P. americana* 02 (Pa02), and *P. brasiliensis* 18 (Pb18).

A Venn diagram was employed to identify overlapping miRNAs (Fig. 1). The miR-92a and miR-941 were common between human lung cells infected with Pb18 or Pa02, while miR-26b-5p, miR-379-5p, and miR-185-5p were common and down-regulated in human lung cells infected with Pl01 or Pb18.

**Fig. 2:**
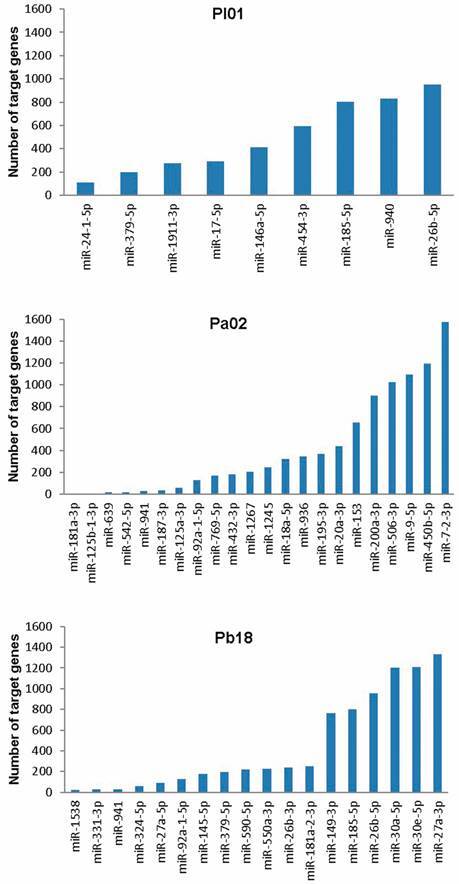
quantity of predicted target genes. Number of target genes for differentially expressed miRNAs in human lung cells infected with *Paracoccidioides lutzii* (Pl01), *P. americana* 02 (Pa02), or *P. brasiliensis* 18 (Pb18).

After, we performed *in silico* analysis of the possible pathways regulated by the set of miRNAs in each case. Gene targets analysis for the differentially expressed miRNAs in the cells infected with each species of *Paracoccidioides* was performed using DIANA miRNA and miRPath tools. The number of predicted target genes varied from 109 (miR-24-1-5p) to 953 (miR-26b-5p) in the cells infected with *P. lutzii*; from 0 (miR-181a-3p) to 1574 (miR-7-2-3p) in the cells infected with *P. americana*; and from 22 (miR-1538) to 1330 (miR-27a-3p) in the cells infected with *P. brasiliensis* (Fig. 2).

Subsequently, pathway analysis considering specifically the cell infection processes demonstrated that although miRNAs identified in the lung cells were different according to the species studied, the potential pathways regulated by miRNAs were common. The identified pathways were related to the process of adhesion between the fungus and the cell (focal adhesion, Rap1 signaling pathway, adherens and GAP junctions); the invasion process (endocytosis and rearrangement of the actin cytoskeleton); the immune response (T cell receptor, Wnt and MAPK signaling pathways), and the induction of apoptosis (MAPK, p53, TGF β and Hippo signaling pathways) (Table IV).

**Fig. 3:**
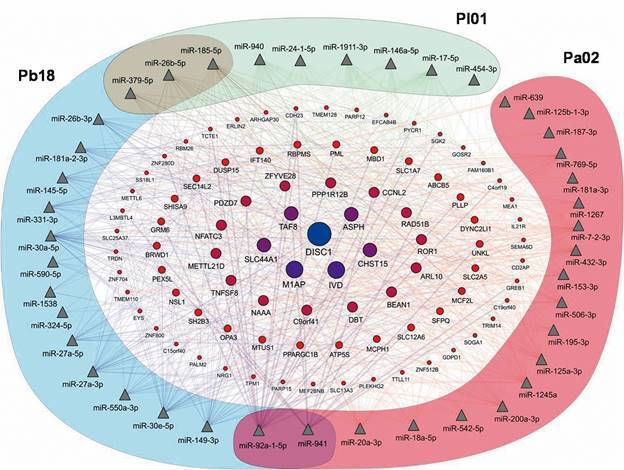
miRNA-target genes interaction network. Triangles represent miRNA and circles represent predicted target genes in human lung cells infected with *Paracoccidioides lutzii* (Pl01), *P. americana* 02 (Pa02), or *P. brasiliensis* 18 (Pb18). The size of the circles is proportional to the number of miRNAs that regulate the genes.

In addition, we found that 89 predicted genes could be targeted by eight or more miRNAs from the different studied species (Fig. 3). Among them, CDH23, CD2AP, and TPM1 could be related to fungus adhesion and invasion to cell, while CCNL2, RAD51B and BRW1 could have a role in apoptosis, and TNFSF8, NFATC3, UNKL, SH2B, EFCAB4B, IL21R, and TRIM14 could be related to the immune response.

**TABLE IV:** Pathways related to the differentially expressed miRNAs in human lung cells infected with *Paracoccidioides* spp.

Pathways	*P. lutzii* 01		*P. americana* 02		*P. brasiliensis* 18	
	Number of miRNAs	p value	Number of miRNAs	p value	Number of miRNAs	p value
Pathways in infection						
Adherens junction	8	0.000107	14	0.000000477	-	
Bacterial invasion of epithelial cells	9	0.0000334	15	0.00835	-	
Endocytosis	9	0.0135	16	0.000232	-	
Focal adhesion	-		16	0.00000836	16	0.00183
Gap junction	8	0.0163	14	0.000233	-	
Phosphatidylinositol signaling system	7	0.0134	15	0.00134	-	
Regulation of actin cytoskeleton	8	0.00107	16	0.000192	13	0.000512
Cell signaling pathways						
cAMP signaling pathway	-		16	0.000917	13	0.0279
ErbB signaling pathway	9	0.00120	14	0.000186	13	0.00164
Hippo signaling pathway	9	0.0165	15	0.00000309	14	0.000384
MAPK signaling pathway	-		20	0.0287	16	0.00406
mTOR signaling pathway	7	0.0482	16	0.0275	-	
p53 signaling pathway	9	0.00693	-		-	
Rap1 signaling pathway	-		16	0.000000477	14	0.00870
T cell receptor signaling pathway	-		17	0.0189	14	0.0174
TGF-beta signaling pathway	7	0.000989	16	0.00000161	15	0.00102
Wnt signaling pathway	8	0.0325	17	0.00000668	16	0.0219
Pathway in amino acid metabolism						
Lysine degradation	8	0.00189	14	0.0189	10	0.00870

## DISCUSSION

MiRNA-mediated gene regulation is remarkable in many infectious diseases and affects cell processes by either mRNA degradation or translational inhibition of proteins.^6^ ^,^ ^7^ Recent studies have focused on the role of miRNAs of host cells after fungal exposure and this group of regulators can influence host responses through a variety of cellular mechanisms.^18^

Lung epithelial cells are the first line of defense of the host against inhaled *Paracoccidioides* spp., but the molecular pattern of these cells in response to the fungi remained unclear. Therefore, this study aimed to evaluate the miRNA profile and the potential pathways they regulate in lung cells exposed to *Paracoccidioides* spp. Surprisingly, our results showed that miRNAs expressed in lung cells were different according to the species of the fungus. Furthermore, most miRNAs were downregulated in the cells infected with *P. lutzii*, while most miRNAs were upregulated in response to *P. americana*. In the cells infected with *P. brasiliensis*, both upregulated and downregulated miRNAs were observed. The host miRNA expression signature as a response to *Paracoccidioides* spp. underlies the elaborate and distinct mechanism of regulation of each species. This species-specific response implies the development of more specific diagnoses and treatments for PCM.^19^ Regarding the use of miRNAs as therapeutic options, one strategy is the use of antisense oligonucleotides (antagomir) to directly block the expression of a disease-associated signature miRNA, which may be relevant for the upregulated miRNAs of lung cells in response to *P. americana* and *P. brasiliensis*. Another strategy could be the use of synthetic miRNA (miRNA mimics) to restore downregulated miRNA expression, as in the case of downregulated miRNAs of lung cells in response to *P. lutzii* and *P. brasiliensis*.

Our results also show that only five miRNAs overlapped, as demonstrated in the Venn diagram. In this context, the comparative analysis of human lung cells exposed to Pb18 or Pa02 identified miR-92a and miR-941 as being common between the two fungal infections. On the other hand, a set of three miRNAs (miR-26b-5p, miR-379-5p, and miR-185-5p) was common and downregulated in host cells infected with Pl01 or Pb18. These miRNAs have also been examined in lung cells or tissue exposed to different pathogens or injuries. Their differential expression and roles described in previous studies allow comparisons to be made and provide future direction in relation to infection by *Paracoccidioides* spp. For example, miR-92A was found to be upregulated in the lung tissues of 57BL/6 mice infected with *A. fumigatus*.^20^ Another study demonstrated the miR-26b is differentially expressed in A549 cells infected with respiratory syncytial virus.^21^ Interesting, miR-26b-5p has also been detected as differentially expressed in the lungs of Balb/c mice infected with *P. brasiliensis* 18, which is in agreement with the present study. Furthermore, a previous study suggests a possible role of miR-26b-5p in the host immune response through the regulation of interleukin (IL)-6 expression.^12^ Regarding miR-185, a previous study showed that it is differentially expressed during oxidative stress in lung epithelial cells with injuries, and induces Bak-dependent apoptosis.^22^ This signaling pathway is also stimulated by *Paracoccidioides* complexes in lung cells,^17^ ^,^ ^23^ suggesting a possible regulatory role played by miRNA-186. On the other hand, in our study, miR-185, miR-379, and miR-941 were first reported to be associated with a lung fungal infection. Therefore, these miRNAs could play important roles and be potential biomarkers in PCM, contributing to species-specific and rapid detection of the infection.

The fact that the miRNA profile in lung cells is different, depending on the fungal species to which it has been exposed, may be due to the *Paracoccidioides* species showing variation in epidemiology, virulence, adaptation, and induction of the immune response in the host cells.^2^ ^,^ ^24^ ^,^ ^25^ ^,^ ^26^ Therefore, we decided to perform *in silico* analysis of the possible pathways regulated by the set of miRNAs in each case. We verified that although miRNAs identified in the lung cells were different according to the species studied, the potential signaling pathways regulated by miRNAs were found to be common. The identified pathways were related to the process of adhesion between the fungus and cells, the invasion process, the immune response, and the induction of apoptosis.

Previous studies have shown that the adhesion process of *Paracoccidioides* spp. to host extracellular matrix components such as collagen type I and IV, laminin, and fibronectin is a crucial step in the interaction.^23^ ^,^ ^27^ ^,^ ^28^ In addition, differences in the adhesion process between phylogenetic species of *Paracoccidioides* to A549 cells have been observed. For example, *P. brasiliensis* 18 has shown a higher capacity for adhesion to cells and adhered more to fibronectin compared to *P. lutzii*, which adhered more to collagen type I and IV.^29^ Consistent with these data, the present results demonstrate that most miRNAs can be associated with important early processes of PCM pathogenesis, such as focal adhesion, bacterial invasion of epithelial cells, Rap1 signaling pathway, adherens, and GAP junctions in A549, in response to *Paracoccidioides*. However, cells infected with *P. lutzii* presented adherens junctions and bacterial invasion of epithelial cells as the most significant pathways, while cells infected with *P. americana* 02 presented adherens junctions, the Rap1 signaling pathway, and focal adhesion, and cells infected with *P. brasiliensis* 18 presented only focal adhesion.

After adhesion, the entry of *P. brasiliensis* in A549 and Vero epithelial cells involves the rearrangement of cytoskeletal components of the host cells, such as actin and tubulin, with the intention of inducing pseudopodia.^23^ ^,^ ^27^ Once inside the host cells, pathogens favor their survival and dissemination through the induction of apoptosis of the system immune cells.^30^ The *Paracoccidioides* complex induces apoptosis in macrophages and lung cells to modify the expression of caspases, Bak, and Bcl 2, and consequently promotes DNA fragmentation.^17^ ^,^ ^23^ ^,^ ^31^ Similarly, our results demonstrate that most miRNAs in the lung cells infected with each of the three species of *Paracoccidioides* may be related to the rearrangement of the actin cytoskeleton and apoptosis through the MAPK, p53, TGF-β, and Hippo signaling pathways.

Finally, we identified miRNAs that may be related to the immune response and inflammation, such as T cell receptor, Wnt, MAPK, and TGF-β signaling pathways. According to previous studies, dendritic cells and B lymphocytes stimulate T cells, which have an important role in the control of PCM.^32^ ^,^ ^33^ The induction of IL, cytokine, and TGF-β levels in host cells was also observed in the interactions with *Paracoccidioides* spp.^1^ ^,^ ^12^ Maza et al. also described that *P. brasiliensis* is able to activate the MAPK pathway in A549 cells, which is involved in the cytokine release by these cells as response to fungal infection.^34^ In addition, the presence of inflammation and formation of granulomas occur in the lung tissue caused by *P*. *brasiliensis* infection.^35^ In the alveolar space, crosstalk between alveolar macrophages and epithelial cells can contribute to host defense. One of the molecular mechanisms that can influence this interaction is the cell-cell release of exosomal miRNAs during infections.^36^ ^,^ ^37^ ^,^ ^38^ Therefore, we believe that in addition to the miRNAs found in the lung epithelial cells, target genes involved in the response to fungi adhesion and invasion, some miRNAs can be exported to regulate genes in neighboring immune cells, contributing to the inflammatory process that occurs in PCM. Similar to the potential involvement of miRNAs in the immune response to three different species of *Paracoccidioides* in our *in vitro* model, a previous study demonstrated a set of differentially expressed miRNAs possibly involved in the *in vivo* immune response by analysing lungs from mice infected with *P. brasiliensis*.^12^ However, further studies are needed to validate this hypothesis.

*In conclusion* - This study provides the first report, to our knowledge, that identifies the miRNAs differentially expressed in human lung cells during *Paracoccidioides* spp. infection. Interestingly, each species (*P. brasiliensis*, *P. americana*, and *P. lutzii*) elicited different expression profiles of miRNAs in the host cells. The results highlight the potential for detecting species-specific infections in *Paracoccidioides* spp., which could be useful in the diagnosis and treatment of PCM. On the other hand, the analysis of predicted targets and potential pathways suggests that the set of miRNAs from three different *Paracoccidioides* species stimulates similar physiological and metabolic processes, which are related to fungi adhesion and invasion and the host immune response. Further studies of validation of miRNAs and their predicted target genes found in the host cell in response to *Paracoccidioides* spp. could contribute to a better understanding of their role in the mechanisms of pathogenesis as well as to the development of a biomarker of PCM.
